# Minimally invasive and full sternotomy in aortic valve replacement: a comparative early operative outcomes

**DOI:** 10.11604/pamj.2021.40.68.28008

**Published:** 2021-09-30

**Authors:** Issaka Zallé, Moussa Son, Mohamed El-Alaoui, Macédoine Nijimbéré, Drissi Boumzebra

**Affiliations:** 1 Cardiovascular Surgery Department, Mohammed VI University Hospital, Marrakech, Morocco

**Keywords:** Minithoracotomy, ministernotomy, sternotomy, aortic valve surgery, early outcomes

## Abstract

**Introduction:**

aortic valve replacement is usually performed through a median full sternotomy (MFS) in our department. Minimally invasive aortic valve replacement (MIAVR) has been recently adopted as a new approach. According to the literature, the superiority of MIAVR is controversial. In this study we report early post-operative outcomes in MIAVR compared with MFS access with reference to blood Loss, wound infections, post-operative recovery, morbidity and mortality.

**Methods:**

this study was a prospective data collection from 36 consecutive patients undergoing isolated valve replacement. Two population study was identified, MIAVR group (group I n=18) and MFS group (group II n=18). Patients´ data were collected and analyzed using IBM SPSS statistics 21 software and Khi2 test has been used to compare the variables. The study variables are presented as numbers, percentage, median with interquartile range. Pre-operative planning was performed so that to obtain similar characteristics.

**Results:**

in group I, upper mini-sternotomy was used in 12 patients and right mini-thoracotomy in 6 patients. There was no difference in term of mortality and morbidity. MIAVR was associated with longer CPB time (93.25 (58-161) vs 131 (75-215) mins, P=0.047) with no significant difference in term of ACC time (81 (33-162) vs 58.8 (59-102) mins P=0.158). MIAVR´ Patients had likely lower incidence of red blood cells transfusion (16.7 vs 52.3%) without significant difference about post-operative haemoglobin (P = 0,330). Patients in group I had shorter ventilation time (2.35 (1-12) vs 9.3 (1-48) hours P < 0.01), shorter ICU stay (2.44 (1-8) vs 4.25 (1-9) days, P = 0,024). The length of hospital stay was shorter, 6.5 (5-9) days in group I vs 7.4 (6-11), P=0.0274. Length of chest tube stay was shorter in group I (mean 1.53 vs 2.4 days, P=0,033). Wound infections were not found in both groups.

**Conclusion:**

minimally invasive aortic valve replacement is associated with less blood loss, faster post-operative recovery faster post-operative recovery but increase operation time.

## Introduction

Aortic valve replacement (AVR) through a full sternotomy (FS) is the traditional standard approach in treatment of aortic valve disease [1]. Minimally invasive aortic valve surgery (MIAVS) has been reported with operative benefits while compared with full sternotomy access [2]. However, some studies have not demonstrated a beneficial effect for minimally aortic valve surgery (MIAVS) [3]. The aim of this study was to report early post-operative outcomes in minimally aortic valve replacement (MIAVR) compared with the access by full sternotomy in order to establish the interest of minimally invasive approach. Our study was based on assessment of blood loss, wound infections, post-operative recovery, morbidity and mortality.

## Methods

**Study design and setting:** this study was a prospectively collected data. It was lead in the Cardiovascular Surgery Department at the Mohammed VI university Hospital, Marrakech, Morocco. This department is a reference hospital for cardiovascular surgery in Morocco in which adults and congenital heart surgery are routinely performed. Among the adult patients admitted to this department for heart valve surgery, a total of 36 patients planned for isolated aortic valve surgery were prospectively selected to perform this study. Assessment of blood loss, wound infections, post-operative recovery, morbidity and mortality related to both surgical approaches was performed.

**Study population:** a critical patient´s selection was used. Our exclusion criteria were emergency cases, patients with aortic root dilation, patients with an unfavorable anatomy, and serious lung disease. Patients requiring concomitant procedures such as coronary artery bypass grafting, or other valves surgery were excluded. The main inclusion criteria were patients with isolated valve replacement. Pre-operative planning included clinical assessment, chest X-ray, transthoracic echocardiography (TTE), and electrocardiogram (ECG) assessment. Coronary angiography was done in the patients with heart risks factors. Peripheral femoral doppler was also performed. Pre-operative planning was performed so that to obtain similar population studies between the two groups. There were no statistical differences between both groups in terms of demographic data, cardiac status, and associated pathologies.

**Surgical technique:** our techniques have been well described in the literature [4-8]. In the group I patients, two different minimally invasive approaches were used in our series: a mini thoracotomy and an upper mini-sternotomy (UMS). About right mini-thoracotomy (RMT), a 4 cm to 6 cm skin incision was placed at the level of the third intercostal space. Five-mm endoscopic trocars were introduced through the fourth and sixth intercostal space at the level of the anterior axillary line; another one in the fifth intercostal space at the level of the middle axillary line in order to perform aortic cross clamping and camera utilization. Femoral-femoral cardiopulmonary bypass (CPB) was performed via a small groin incision. Most cases used mild hypothermia to normothermia CPB. The ascending aorta is clamped with a low-profile aortic cross clamp and antegrade cold blood cardioplegia is delivered directly. The aortic valve is replaced as usual fashion. The upper mini-sternotomy (UMS) is achieved through 6 to 10 cm midline vertical skin incision, performing a partial J sternotomy at the third to fifth intercostal space. Standard cannulation of the ascending aorta was performed directly through the incision. Venous cannulation was performed either via the right atrial appendage in many of our patients or with femoro-femoral cannulation. A transverse aortotomy is placed higher. For the group II patients, a full median sternotomy was performed and aortobicaval cannulation for CPB using normothermia in most cases.

**Data collection:** a prospectively collected data was analysed from November 2017 to October 2019 using IBM SPSS statistics 21 software. The patients were divided into two groups, the group I (n=18) underwent MIAVR and in the group II (n=18) was operated with MFS. The decision to perform MIAVS or FS depended to the surgeons. Peri-operative and 30 days follow-up data were collected and compared.

**Definitions:** the variables like Length of chest drain stay, intensive care unit (ICU) stay, ventilation time, cardiopulmonary bypass time, aortic cross clamp time, wound infections were appreciated in term of comparison in reference to P value. Blood loss was defined as the difference of pre-operative and post-operative hemoglobin and the incidence of blood transfusion.

**Statistics analysis:** continuous variables were collected and described by the median with interquartile range, and other variables using their numbers, frequencies and percentages. The data were compared using Khi2 test, P<0.05 is considered statically significant with confidence interval at 95%.

**Ethical considerations:** individual consent based by oral request was obtained from all patients before the surgery. The patients have been informed about the risks of surgery and the interest of the study.

## Results

The following variables are presented as numbers, percentage, median with interquartile range. Pre-operative data (Table 1) shown that the mean age of the patients was 48.1 (28-69) years in the MIAVR group and 56.3 (18-84) years in the MFS group, P=0.315. Sex ratio 3.25 (group I vs 2 group II, P=0.485); the mean weight was 63.8 (56-82) kg in the group I, 62.7 (40-90) kg in the group II, P=0.480. Left ventricular ejection fraction (LVEF) was 58.3% (35-73) in the group I vs 51.7% (25-78), P=0.359. This characteristics baseline did not show significantly difference in the two groups. The mean pre-operative hemoglobin was not different in both groups (13.9 (10.953-15.3) in group I vs 13.4 (7.8-16) g/dl in group II, P=0,444). All the patients underwent AVR; any patient in the MIAVR was required reconversion to full sternotomy. An upper mini-sternotomy approach was used in 12 and six (6) RAMT were performed. One (1) patient received a bioprosthetic valve (bovine pericardial). All procedures were uneventful.

**Table 1:** pre-operative variables and data analysis

Variables	Minimally invasive	Full sternotomy	P value
Number	18	18	
Age, mean and range	48.1 (28-69)	56.3 (18-87)	0.3156
Sex ratio	3.25	2	0.0485
Weight (kg)	63.8 (56-82)	62.7 (40-90)	0.4801
Arterial hypertension (%)	11.1	23.8	
Mellitus diabetes (%)	16.67	19	
History of smoking (%)	16.67	19	
Dyslipidaemia (%)	11.1	9.5	
NYHA, mean and rang	2.4 (1-3)	2.71 (1-4)	0.3192
Aortic stenosis (%)	58	62.5	
Aortic stenosis + aortic regurgitation (%)	29.55	27	
Aortic regurgitation (%)	12.45	10.5	
Post rheumatic (n)	10	14	
degenerative disease (n)	5	3	
Aortic bicuspid (n)	3	1	
LVEF, mean and range (%)	58.3% (35-73)	51.7 (25-78)	0.3594
Haemoglobin, mean and range (g/dl)	13.9 (10.9-15.3)	13.4 (7.8-16)	0.444

NYHA: New York heart association; LVEF: left ventricular ejection fraction

Intraoperative data (Table 2) revealed that FS group had a shorter CPB time (93.25 (58-161) vs 131 (75-215) min, P=0.047), but not significantly aortic cross-clamp time difference (81 (33-162) vs 58.8 (59-102), P=0.158). Examination of postoperative outcomes (Table 2) revealed that MIAVR´ patients had likely lower incidence of red blood cells transfusion (16.7 vs 52.3%) and less requirement inotropic support (16.7 vs 66.7%, P=0.003). Mechanical ventilation time and ICU stay were found to be shorter in the group I respectively (2.35 (1-12) vs 9.3 (1-48) hours, P<0.01); (2.44 (1-8) vs 4.25 (1-9) days, P=0.024). The chest tube was removed earlier in the group I (mean 1.53 vs 2.4 days, P=0.0274). There was no significant difference about post-operative haemoglobin. Haemorrhage complications were more likely in the group II than the group I (44.4 vs 16.7%). The incidence of pulmonary complications was similar in both groups. In contrast to the group II, cerebrovascular events in one patient were found in the group I. The length of hospital stay was 6.5 (5-9) days in the group I vs 7.4 (6-11) in group II, P=0.033. In both groups, there was no in-hospital and 30-days mortality.

**Table 2:** intra and post-operative variables and data analysis

Variables	Minimally invasive	Full sternotomy	P value
CPB time, mean and range (mins)	131 (75-215)	93.25 (58-161)	0.0475
ACC time, mean and range (mins)	81 (33-162)	58.8 (59-102)	0.1587
Red blood cells transfusion (%)	16.7%	52.3%	
Haemoglobin, mean and range (g/dl)	11.78 (7.3-14.4)	10.3 (7.9-12.9)	0.330
Inotropic using (%)	16.7	66.7	
Inotropic duration, mean and range (days)	1.25 (1-3)	2.3 (1-4)	0.0036
Ventilation time, mean and range (hours)	2.35 (1-12)	9.3 (1-48)	<0.0001
Length of chest drain stay	1.53 (1-2)	2.4 (2-5)	0.0274
Pulmonary complications (%)	5.5	5.5	
Cerebrovascular events (%)	5.5	0	
Haemorrhage complications (%)	16.7	44.4	
ICU stay, mean and range (days)	2.44 (1-8)	4.25 (1-9)	0.0244
LVEF, mean and range (%)	54.4 (30-76)	51 % (20-78)	0.4286
Mean aortic gradient mean and range (mmHg)	10.22 (5-19)	10.5 (5-23)	0.4721
Normal function of prosthesis (%)	100	100	
Mechanical prosthesis (%)	100	100	
Aortic repair (n)	0	0	
Wound infections (n)	0	0	
Bioprosthesis (n)	1	0	
Reconversion to full sternotomy (%)	0	-	
Length of hospital stay (mean and range)	6.5 (5-9)	7.4 (6-11)	0.033
In hospital mortality (%)	0	0	

CPB: cardiopulmonary bypass; ACC: aortic cross-clamp; ICU: intensive care unit; LVEF: left ventricular ejection fraction

## Discussion

We did not find any difference in term of mortality, morbidity in our series. However, MIAVR was associated with lower incidence of blood loss; shorter mechanical ventilation time, intensive care unit stay, and length of hospital stay compared with the group undergoing full sternotomy. Moreover, faster recovery was associated with MIAVR versus group of FS. The group of FS had a shorter CPB time but not significantly difference in view of aortic cross-clamp (ACC) time. After the first valvular surgery through right thoracotomy under CPB [9], FS approach was adopted as a gold standard and make safely surgical procedures. That conventional approach proved long-term success in aortic and mitral valve surgery [10]. Otherwise MIAVR has been reported with superior benefits while compared with FS access [2]. According to the literature the two surgical procedures can be compared with reference to certain operative variables.

In fact, our series did not show any difference in term of morbidity and mortality. Our observations are consistent with Glauber *et al*. [11], Del Giglio *et al*. [12] who showed any difference between MIAVS and FS in term of peri-operative morbidity and mortality. Merk *et al*. [13] reported the same results but with a higher long-term survival in MIAVS than the group of FS. However, as our series MIAVS is associated with improve early post-operative outcomes compared with FS procedure. Glauber *et al*. [11], Merk *et al*. [13] and Bakir *et al*. [14], reported in their studies that MIAVS is associated with shorter ventilation time, length of hospital stays, lower blood loss. According to Glauber *et al*. [11], MIAVR performed through RMT, or UMS is a safe procedure associated with excellent postoperative outcomes in terms of mortality, morbidity, shorter hospital stay and faster recovery. In contrast current studies such as the one of Young *et al*. [15] and Del Giglio *et al*. [12] showed similar outcomes between both procedures. Particularly Young *et al*. [15], had concluded through a literature review that benefits are demonstrable, particularly in higher risk, comorbid settings. Brown *et al*. [16] in meta-analysis had reported that mini-sternotomy can be performed safely for aortic valve replacement, without increased risk of death or other major complication; however, few objective advantages have been shown. Throughout these studies including our study, we can say that minimally invasive (MI) for AVR improves early post-operative outcomes compared with FS access despite some controversy results reported by other studies. Moreover, our series did not find wound infection.

The series of Glauber *et al*. [11], this study reported that the occurrence of stroke, renal failure, reexploration for bleeding and wound infection was similar in both groups. These complications should not relate to the type of procedure. Compared with FS approach, MIAVR is known to provide faster recovery. In addition, MIAVR has shown to improve post-operative respiratory function due to the preservation of sternum and reduction of post-operative pain, blood loss and blood transfusions related to the reduction of surgical dissection.

In our series FS group had a shorter CPB time but not significantly difference in view of aortic cross-clamp time; our observation is consistent with Del Giglio *et al*. [12] and Merk *et al*. [13]. The increased operation time in MI approach is reported by many comparative studies. Despite advantages of MIAVR, it is limited by the longer operative time which may compromise operative outcomes in high operative risk patients. According to the literature, suture less valve could be an alternative to reduce operative time in MIAVS and so allows performing minimally invasive safely in high-risk patients [17-19]. Our study is limited in term of population size; however, it provides a comparative result with many studies cited above. That makes it consistent with the literature.

## Conclusion

Minimally invasive aortic valve replacement is associated with less blood loss, faster post-operative recovery but increase operation time. Our study confirms that minimally approach can be used safely in aortic valve replacement.

### What is known about this topic

- In our countries, aortic valve diseases are frequent and are dominated by rheumatologic aetiology;
- Aortic valve replacement is the main treatment and is routinely performed by way of full stertotomy with improved outcomes;
- Minimally invasive approach is less used in our area but more used in development countries nowadays.

### What this study adds

- Minimally invasive aortic valve replacement is a new surgical approach; according to our study, it can be performed whatever the age of the patient;
- Our study confirms that this approach can be performed safely with improved early post-operative outcomes like fast recovery;
- This study provides a new data of minimally invasive approach in valve surgery.
